# Tirzepatide as a novel effective and safe strategy for treating obesity: a systematic review and meta-analysis of randomized controlled trials

**DOI:** 10.3389/fpubh.2024.1277113

**Published:** 2024-01-31

**Authors:** Wenting Cai, Ruobin Zhang, Yao Yao, Qiuhui Wu, Jinping Zhang

**Affiliations:** ^1^Department of Pharmacy, Nanjing Drum Tower Hospital, School of Basic Medicine and Clinical Pharmacy, China Pharmaceutical University, Nanjing, China; ^2^Department of Pharmacy, Nanjing Drum Tower Hospital, Affiliated Hospital of Medical School, Nanjing University, Nanjing, China

**Keywords:** overweight, obesity, BMI, waist circumference, tirzepatide

## Abstract

**Objective:**

To systematically evaluate the efficacy and safety of a new hypoglycemic drug, tirzepatide, for treating obesity based on indicators such as BMI, waist circumference, and body weight.

**Methods:**

A search formula was written using search terms such as “tirzepatide,” “overweight,” and “obesity.” A comprehensive search was conducted on databases such as PubMed, Cochrane Library, Embase, and Web of Science using a computer. Random controlled trial (RCT) literature was selected based on inclusion and exclusion criteria. After extracting the data, literature bias risk assessment and meta-analysis were conducted using RevMan 5.4 software. The search deadline is from the establishment of each database to May 2023.

**Results:**

A total of 12 randomized controlled trials were included, with a total of 11,758 patients. Meta analysis results showed that compared with the glucagon like peptide-1 receptor agonist (GLP-1 RAs), placebo and insulin groups, tirzepatide could significantly reduce the BMI (body mass index) of patients [MD = −1.71, 95% CI (−2.46, −0.95), *p* < 0.00001], [MD = −3.99, 95% CI (−3.69, −2.45), *p* < 0.00001], [MD = −4.02, 95% CI (−4.72, −3.31), *p* < 00.00001]. In terms of decreasing waist circumference, tirzepatide has a more significant advantage [MD = −4.08, 95% CI (−5.77, −2.39), *p* < 0.00001], [MD = −7.71, 95% CI (−10.17, −5.25), *p* < 0.00001], [MD = −9.15, 95% CI (−10.02, −8.29), *p* < 0.00001]. In the analysis of body weight, tirzepatide showed a more significant reduction effect compared to the control group [MD = −5.65, 95% CI (−7.47, −3.82), *p* < 0.001], [MD = −10.06, 95% CI (−12.86, −7.25), *p* < 0.001], [MD = −10.63, 95% CI (−12.42, −8.84), *p* < 0.001]. In comparison with placebo, tirzepatide had a prominent advantage in weight loss ≥20% and ≥25% [RR = 30.43, 95% CI (19.56, 47.33), *p* < 0.00001], [RR = 37.25, 95% CI (26.03, 53.30), *p* < 0.00001]. Subgroup analysis showed a dose-dependent therapeutic effect. In terms of safety, compared with the placebo and insulin groups, the incidence of gastrointestinal adverse reactions was markedly higher in the tirzepatide group, slightly higher to the GLP-1 RAs group. The hypoglycemic (<70 mg/dL) risk of tirzepatide was slightly higher to that of placebo and GLP-1 RAs, but significantly lower than that of the insulin group [RR = 0.46, 95% CI (0.36, 0.58), *p* < 0.001]. The incidence of other adverse events, including pancreatitis, cholecystitis, major adverse cardiovascular events-4, hypersensitivity reactions, and neoplasms did not show significant statistical differences compared to the control group (*p* > 0.05).

**Conclusion:**

Tirzepatide, as a weight loss drug, significantly reduces BMI, waist circumference and body weight while gastrointestinal adverse reactions need to be vigilant. Overall, its efficacy is significant and its safety is high.

## Introduction

Obesity is not only a chronic metabolic disease, but also a major public health issue. In the past 50 years, the number of people worldwide suffering from obesity has tripled, with over 650 million adults considered obese and at least 1.9 billion adults overweight (1, 2). In the past 40 years in China, the number of overweight and obese individuals has also rapidly increased. According to statistics, from 2015 to 2019, the estimated prevalence of overweight and obesity among adults (≥18 years old) in China reached 34.3 and 16.4%, and even among children and adolescents aged 6–17, the prevalence of overweight and obesity reached 11.1 and 7.9%, respectively (3). Many large-scale population studies have reported a BMI exceeding 30 kg/m^2^ (defined as obesity in many guidelines), which is significantly associated with an increased risk of morbidity and mortality (4–6). In 2015, 4 million deaths were caused by high BMI, of which over two-thirds were caused by cardiovascular disease (7). Therefore, overweight and obesity have become the main risk factors for cardiovascular disease. BMI and waist circumference are also closely related to T2DM and cardiovascular disease (8–11). Weight loss can reduce the risk of cardiovascular disease and the incidence of diabetes (12).

More and more drugs such as GLP-1 RAs have been proven to be used as weight loss drugs. Liraglutide is the first GLP-1 RAs approved by the US Food and Drug Administration (FDA) and the European Medicines Agency (EMA) for the treatment of obesity (13). In recent years, significant progress has been made in the research and development of GLP-1 RAs. Due to their effective hypoglycemic efficacy and significant weight loss effects, multiple drugs have been approved for marketing and widely used (14).

In May 2022, the hypoglycemic drug tirzepatide (trade name: Mounjaro) developed by Eli Lilly and Company was approved by the FDA for marketing. This is the first dual agonist of glucose dependent insulinotropic peptide (GIP) and GLP-1 receptor (15). GIP inhibits gastric secretion activity, stimulates insulin secretion, has insulin-like effects on adipose tissue, inhibits fat lysis, and promotes fat generation (16, 17). GLP-1 can stimulate insulin secretion and inhibit the release of glucagon. It can also slow down gastric emptying and induce a feeling of fullness (18). Both GIP and GLP-1 belong to the insulin stimulating hormone, and the secretion of these insulin stimulating hormones may be caused by nutrients in the gut, microbial factors, and neuroendocrine stimulation. In turn, GIP and GLP-1 lead to increased insulin secretion and peripheral insulin sensitivity, while slowing the neuroregulation of gastric emptying and gastrointestinal motility (19). Therefore, on the basis of diet control and enhanced exercise, tirzepatide can improve blood sugar level and reduce weight in obese patients with type 2 diabetes (20, 21). Notably, GLP-1 inhibits glucagon while GIP increases, which may produce a good balance for avoiding hypoglycemia (22). In a recent clinical trial, it was shown that tirzepatide can safely reduce BMI, waist circumference and body weight by 20%, and its weight loss effect is far superior to the older generation of weight loss drugs (23). The latest research results released by SURMOUNT-2 (24) also support this conclusion. Tirzepatide is bound to become a strong competitor in the field of weight loss and it can also effectively prevent cardiovascular disease.

This article conducts a comprehensive search of relevant literature both domestically and internationally, and uses meta-analysis to systematically evaluate the included literature, in order to confirm the effectiveness and safety of tirzepatide in treating obesity and provide strong evidence for its approval as a weight loss drug.

## Methods

### Search strategy

We conducted a comprehensive search on databases PubMed, Cochrane Library, Embase, and Web of Science, with a search deadline of May 2023 for each database since its establishment. The following descriptors were used:(tirzepatide OR LY3298176) AND (obese OR obesity OR overweight). Expand the search of the included references in the database.

### Inclusion criteria

(1) Research patients: overweight or obese patients with or without type 2 diabetes mellitus (T2DM), regardless of age, gender, and course of disease. (2) Interventions: patients in the experimental group received tirzepatide, while those in the control group received GLP-1 RAs or placebo or insulin. (3) Outcome indicators: the efficacy indicators mainly include BMI, waist circumference and body weight. The safety indicators mainly include total adverse events (AE), serious adverse events (SAE), gastrointestinal adverse reactions, hypoglycemia (<70 mg/dL) and major adverse cardiovascular events-4 (MACE-4). (4) Research type: only randomized controlled trials. (5) Language type: unlimited. Overweight and obesity in Asians (4) were defined as a BMI of 23–24.9 kg/m^2^ and ≥25 kg/m^2^, while a BMI of 25–29.9 kg/m^2^ and ≥30 kg/m^2^ for Europeans (25). MACE-4 mainly refers to death due to cardiovascular causes, non-fatal myocardial infarction, non-fatal stroke or hospitalization for unstable angina.

### Exclusion criteria

(1) Researches such as review, abstract and report. (2) Not RCT. (3) Animal experiments. (4) Researches with incomplete data. (5) Researches published repeatedly.

### Literature selection and data collection

We used NoteExpress software to manage all literature. After clarifying and unifying the screening criteria, two individuals independently conducted a preliminary screening of all literature. For literature with differing opinions, a third person would read the full text and decide whether to include it. Excel 2019 software was used to extract data. One person entered the data and the other checked it. The extracted content includes the name of the first author, the year of publication, the study style, the study sites, the study population, the intervention measures, the sample size, and the study duration.

### Quality assessment

We assessed the bias risk of the included literature according to the RCT bias assessment tool in Cochrane’s manual. The bias risk assessment comprehensively considered seven aspects, including random sequence generation, allocation concealment, blinding of participants and personnel, blinding of outcome assessment, incomplete outcome data, selective reporting, and other bias. Two individuals independently evaluated the risk of bias in the quality of the literature, and for literature with differing opinions, a third person evaluated it.

### Statistical analysis

We used RevMan 5.4 software for meta-analysis. Continuous variables used mean difference (MD) to analyze the effect size, and two categorical variable used risk ratio (RR) to analyze the effect size. Confidence interval (CI) was set to 95% CI. Chi square (χ^2^) was used to test the statistical heterogeneity between the evaluation results, and use *I*_2_ quantitative judgment for heterogeneity. When *I*_2_ > 50%, there is a statistical difference in the results. Use a random effects model and sensitivity analysis for the source of heterogeneity. When *I*_2_ < 50%, use a fixed effects model. *p* < 0.05 indicates statistical significance for the difference. Conduct subgroup analysis based on the different dosages (5 mg, 10 mg, 15 mg) given to the experimental group.

## Results

### Search results

**Figure 1:**
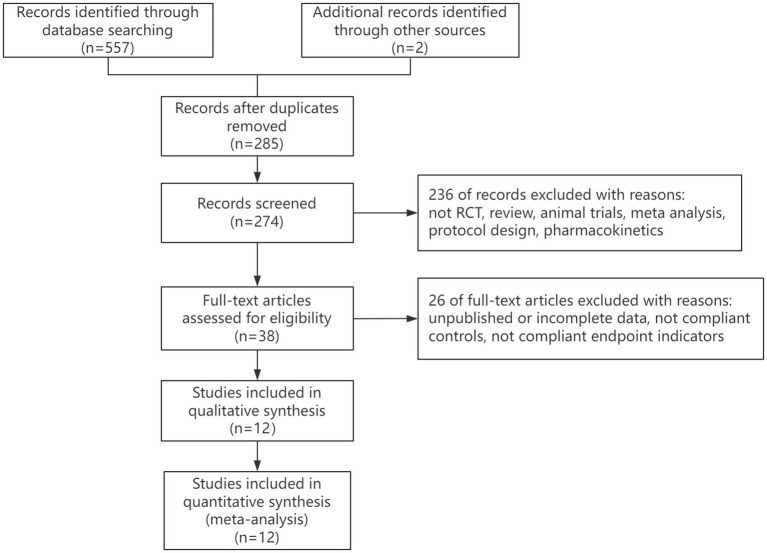
Flow diagram of the selection process of studies.

A total of 559 articles were retrieved in this study, with 285 duplicate articles excluded. 274 articles were included in the initial screening. two hundred and thirty-six articles were excluded by reading the title and abstract of the articles, and 26 articles were excluded by reading the full text of the articles. Finally, 12 articles were included (23, 26–35). The literature retrieval process is shown in Figure 1.

### Study and participant characteristics

As shown in Table 1, the study sites involve a large number of countries. Eleven articles (26–35) were about obese or overweight patients with T2DM, and 1 article (23) was about obese patients without T2DM. The experimental group consisted of tirzepatide, while the control group consisted of GLP-1 RA with 4 cases [2 (26, 34) cases of dulaglutide and 2 (29, 33) cases of semaglutide], 8 (23, 24, 26, 27, 31–34) cases of placebo, and 3 (28, 30, 35) cases of insulin. A total of 11,758 patients were included and the study duration were all ≥12 weeks.

**Table 1:** Study and participant baseline characteristics of included RCTs.

The first author, year published	Study style	Study sites	Study population	Intervention	Sample size	Control	Sample size	Study duration
Frias et al. (26)	Phase 2b, randomized controlled, double-blind	47 research sites in Poland, Puerto Rico, Slovakia, and the United States	18–75 years old, with T2DM for at least 3 months, BMI 23–50 kg/m^2^	Tirzepatide (5, 10, 15 mg)	55, 51, 53	Dulaglutide or placebo	54, 51	26
Frias et al. (27)	Phase 2, randomized controlled, double-blind	13 research points in the United States	With T2DM for at least 3 months, BMI 23–45 kg/m^2^	Tirzepatide (15 mg^−1^, 15 mg^−2^)*	28, 28	Placebo	26	12
Del Prato et al. (28) (SURPASS-4)	Phase 3, randomized controlled, open-label	187 research points in Argentina, Australia, Brazil, Canada, Greece, Israel, Mexico, Poland, Romania, Russia, Slovakia, Spain, Taiwan, and the USA	≥18 years old, with T2DM, BMI ≥25 kg/m^2^	Tirzepatide (5, 10, 15 mg)	329, 328, 338	Insulin	1,000	52
Frías et al. (29) (SURPASS-2)	Phase 3, randomized controlled, open-label	128 research sites in the United States, Argentina, Australia, Brazil, Canada, Israel, Mexico, and the United Kingdom	≥18 years old, with T2DM, BMI ≥25 kg/m^2^	Tirzepatide (5, 10, 15 mg)	470, 469, 470	Semaglutide	469	40
Ludvik et al. (30) (SURPASS-3)	Phase 3, randomized controlled, open-label	122 research sites in Argentina, Austria, Greece, Hungary, Italy, Poland, Puerto Rico, Romania, South Korea, Spain, Taiwan, Ukraine, and the United States	≥18 years old, with T2DM for at least 3 months, BMI ≥25 kg/m^2^	Tirzepatide (5, 10, 15 mg)	358, 360, 359	Insulin	360	52
Rosenstock et al. (31) (SURPASS-1)	Randomized controlled, double-blind	52 research sites in India, Japan, Mexico, and the United States	≥18 years old, with T2DM, BMI ≥23 kg/m^2^	Tirzepatide (5, 10, 15 mg)	121, 121, 121	Placebo	115	40
Dahl et al. (32) (SURPASS-5)	Phase 3, randomized controlled, double-blind	45 research sites in the United States, Japan, Czech Republic, Germany, Poland, Puerto Rico, Slovakia, and Spain	With T2DM, BMI ≥23 kg/m^2^	Tirzepatide (5, 10, 15 mg)	116, 119, 120	Placebo	120	40
Heise et al. (33)	Phase 1, randomized controlled, double-blind	2 research points in Germany	20–74 years old, with T2DM for at least 6 months, BMI ≥25 kg/m^2^	Tirzepatide (15 mg)	45	Semaglutide or placebo	44, 28	28
Inagaki et al. (34) (SURPASS J-mono)	Phase 3, randomized controlled, double-blind	Japan	≥20 years old, with T2DM for at least 8 months, BMI ≥23 kg/m^2^	Tirzepatide (5, 10, 15 mg)	159, 158, 160	Dulaglutide or placebo	159	52
Jastreboff et al. (23) (SURMOUNT-1)	Phase 2, randomized controlled, double-blind	119 research points from 9 countries	≥18 years old, without T2DM, BMI ≥30 kg/m^2^ or ≥ 27 kg/m^2^	Tirzepatide (5, 10, 15 mg)	630, 636, 630	Placebo	643	72
Gao et al. (35) (SURPASS-AP-Combo)	Phase 3, randomized controlled, open-label	66 research points in Asia Pacific regions such as China, South Korea, Australia, and India	≥18 years old, with T2DM for at least 2 months, BMI ≥23 kg/m^2^	Tirzepatide (5, 10, 15 mg)	230, 228, 229	Insulin	220	40
Garvey et al. (24) (SURMOUNT-2)	Phase 3, randomized controlled, double-blind	77 research points in Argentina, Brazil, India, Japan, Russia, Taiwan, and the USA	≥18 years old, with T2DM for at least 3 months, BMI ≥27 kg/m^2^	Tirzepatide (10, 15 mg)	312, 311	Placebo	315	72

^*^Different ways of increasing dose: 15 mg^−1^: 2.5 mg →5 mg →10 mg→15 mg; 15 mg^−2^: 2.5 mg→7.5 mg→15 mg.

T2DM, Type 2 diabetes; BMI, Body mass index.

### Literature quality assessment

**Figure 2:**
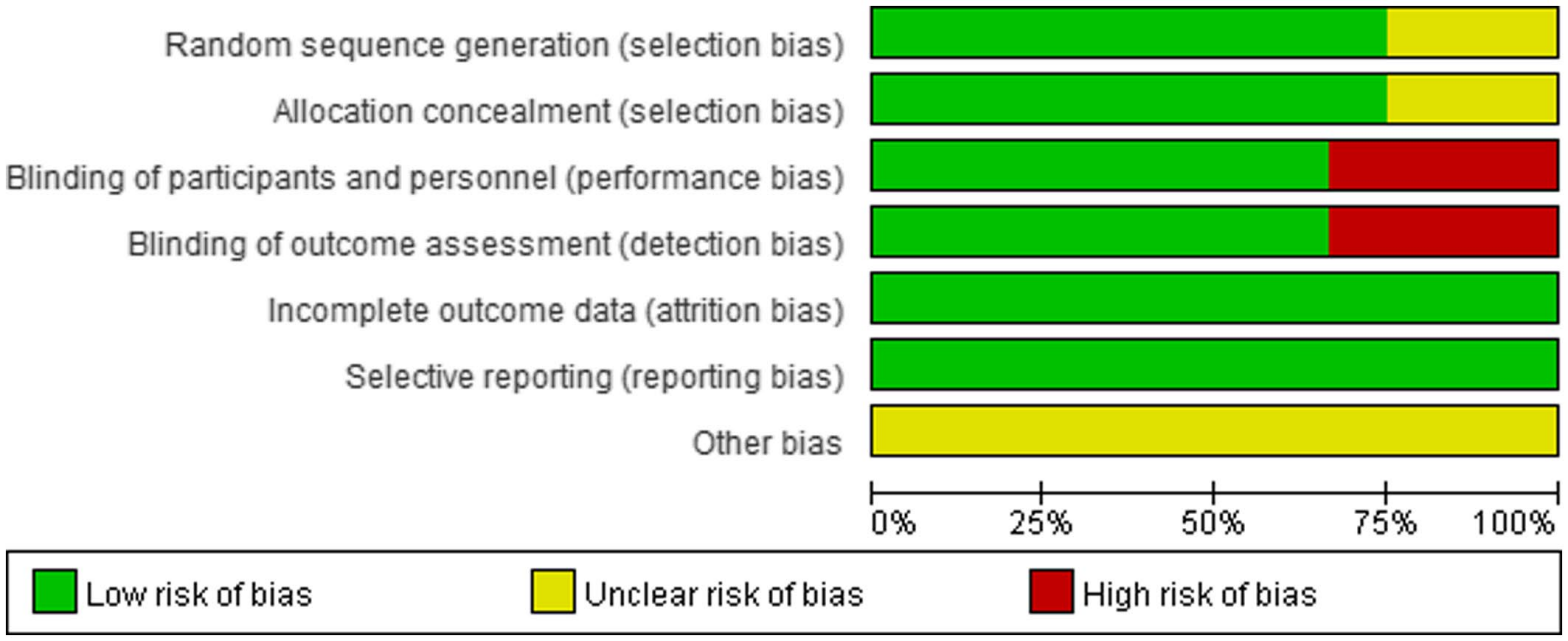
Risk of bias graph for all included studies.

**Figure 3:**
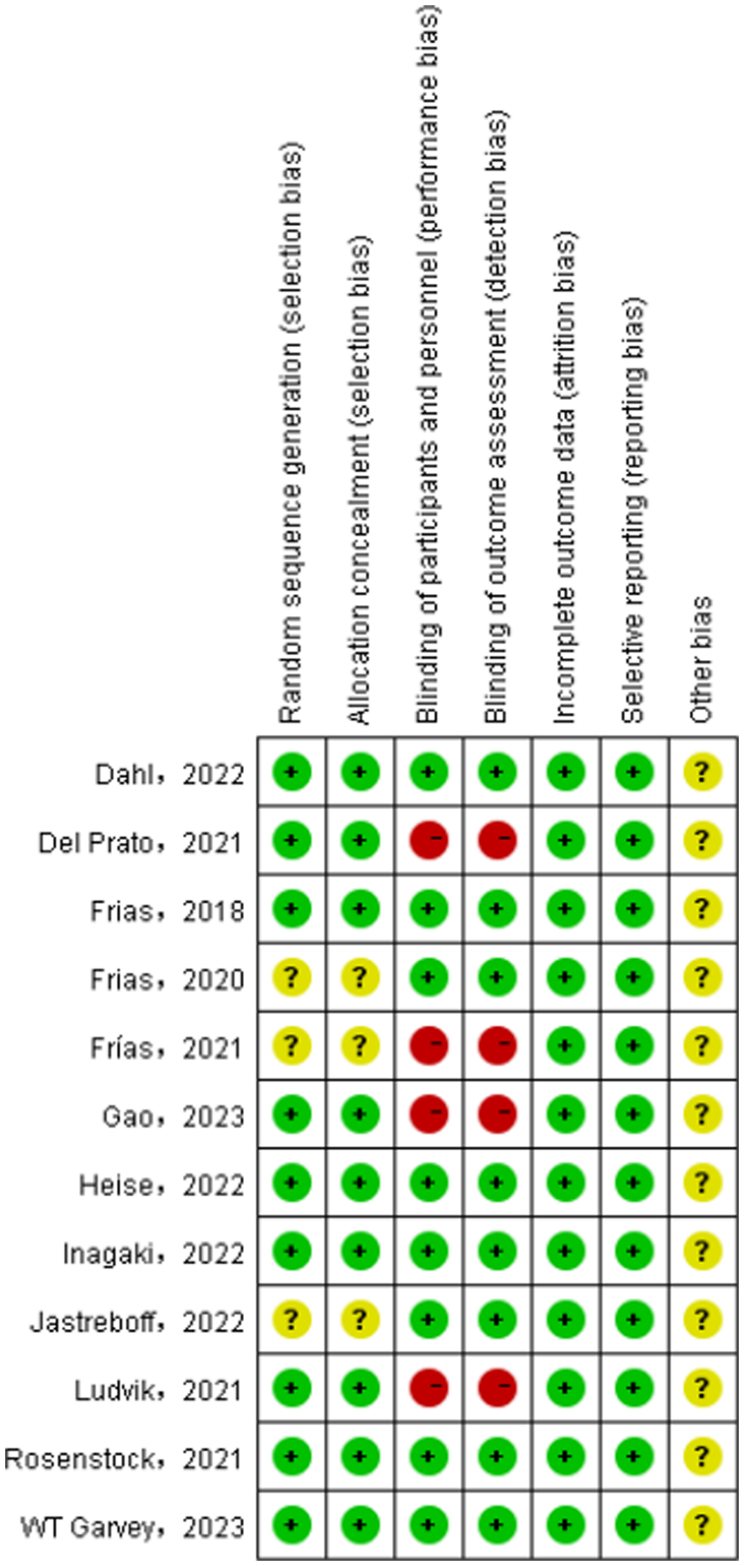
Risk of bias summary for all included study.

As shown in Figures 2, 3, nine studies (24, 26, 28, 30–35) detailed the sequence generation, marked as “low risk,” Three studies (23, 27, 29) did not specify the allocation concealment and marked as “unclear.” Eight RCTs (23, 24, 26, 27, 31–34) were double-blind trials labeled as “low risk,” while four RCTs (28–30, 35) had detection bias labeled as “high risk.” No attrition bias or reporting bias was found, so all RCTs were labeled as “low risk.”

### BMI

**Figure 4:**
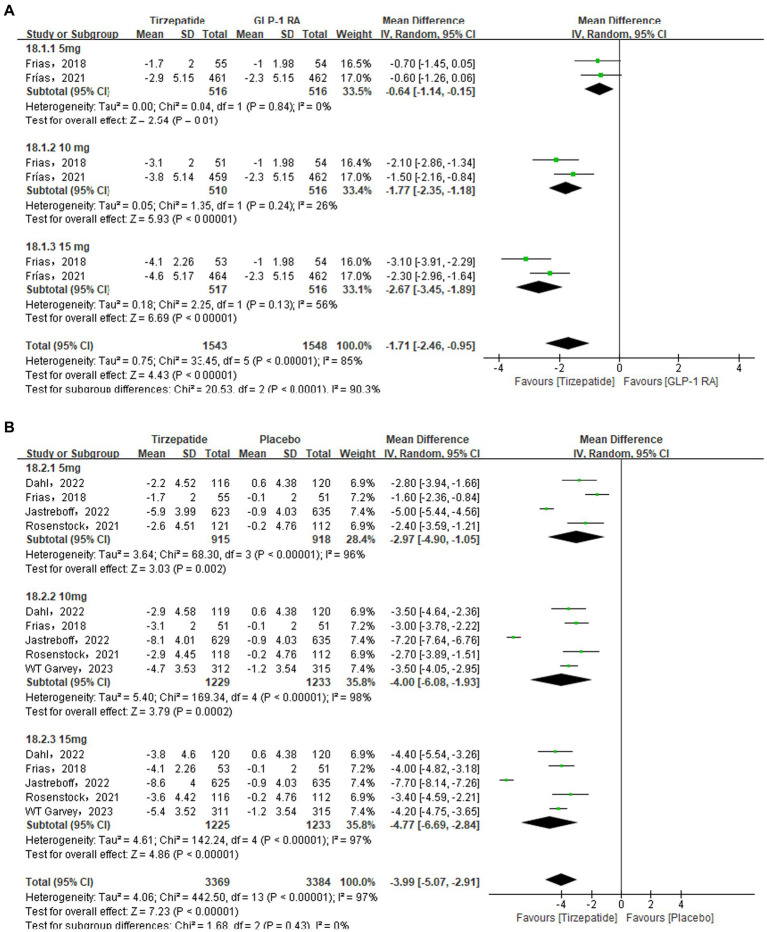

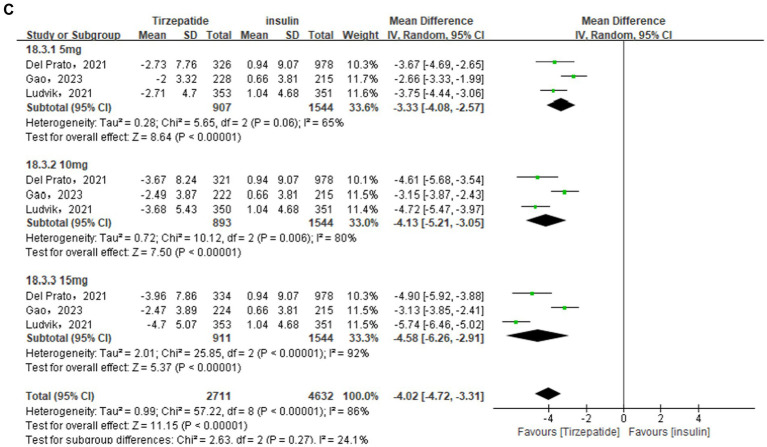
Forest diagram for tirzepatide vs GLP-1 RAs **(A)** and vs. placebo **(B)** and vs insulin **(C)** for change in BMI (kg/m^2^). GLP-1 RA: semaglutide and dulaglutide.

The comparison of the tirzepatide and GLP-1 RA groups used a random effects model, due to the statistical heterogeneity [*I*_2_ = 85%, *p* < 0.00001]. According to the findings of the meta-analysis, the tirzepatide group had lower BMI than the control group [MD = −1.71, 95% CI (−2.46, −0.95), *p* < 0.00001]. Look at Figure 4. Using a random effects model also demonstrated statistical heterogeneity in the comparison between the tirzepatide and placebo groups [*I*_2_ = 97%, *p* < 0.00001]. The meta-analysis results showed that the BMI of the tirzepatide group was significantly lower than that of the control group [MD = −3.99, 95% CI (−3.69, −2.45), *p* < 0.00001]. Using a random effects model in the comparison of the tirzepatide and insulin groups [*I*_2_ = 86%, *p* < 0.00001]. The results of the meta-analysis indicated that the tirzepatide group’s BMI was statistically significant reduced than that the control group’s [MD = −4.02, 95% CI (−4.72, −3.31), *p* < 0.00001]. Each subgroup’s analysis proved that the outcomes were dose-dependent.

Sensitivity analysis was performed owing to the high heterogeneity in the results. When the Jastreboff et al. (23) study in the placebo group and the Ludvik et al. (30) study in the insulin group were excluded, sensitivity analysis found that the overall statistical heterogeneity dropped from 97 to 75%, and from 86 to 74%, respectively. The weight of the tirzepatide group was significantly lower than that of the control group [MD = −3.07, 95% CI (−3.69, −2.45), *p* < 0.00001; MD = −4.02, 95% CI (−4.72, −3.31), *p* < 0.00001]. The results were consistent with those discovered before to the sensitivity analysis, with statistically significant differences and high reliability of the results.

### Waist circumference

**Figure 5:**
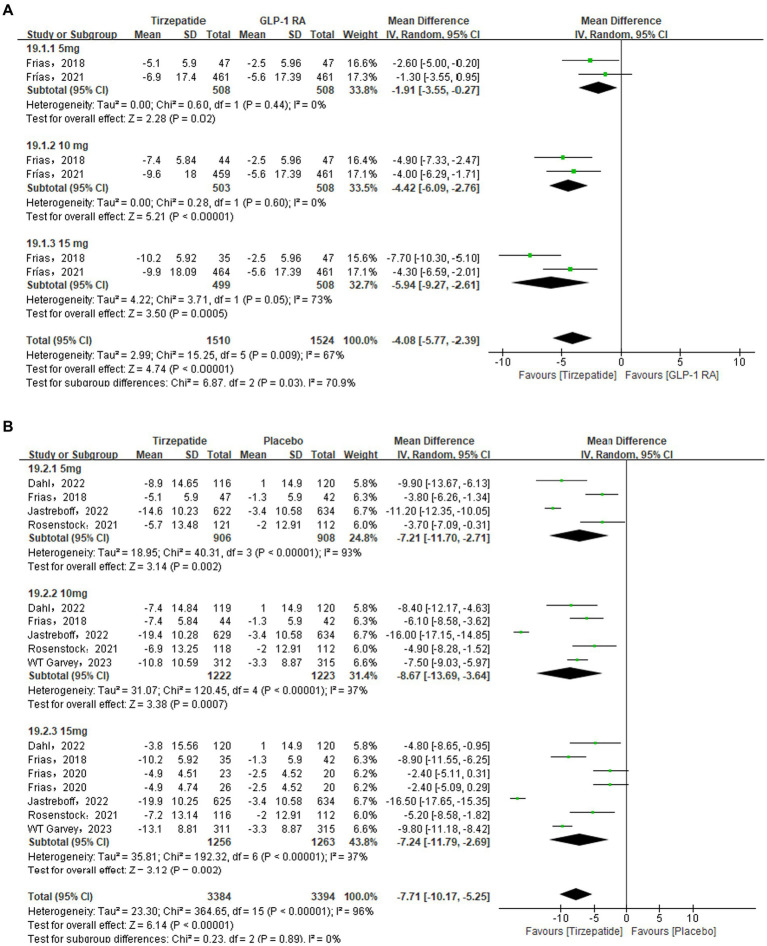

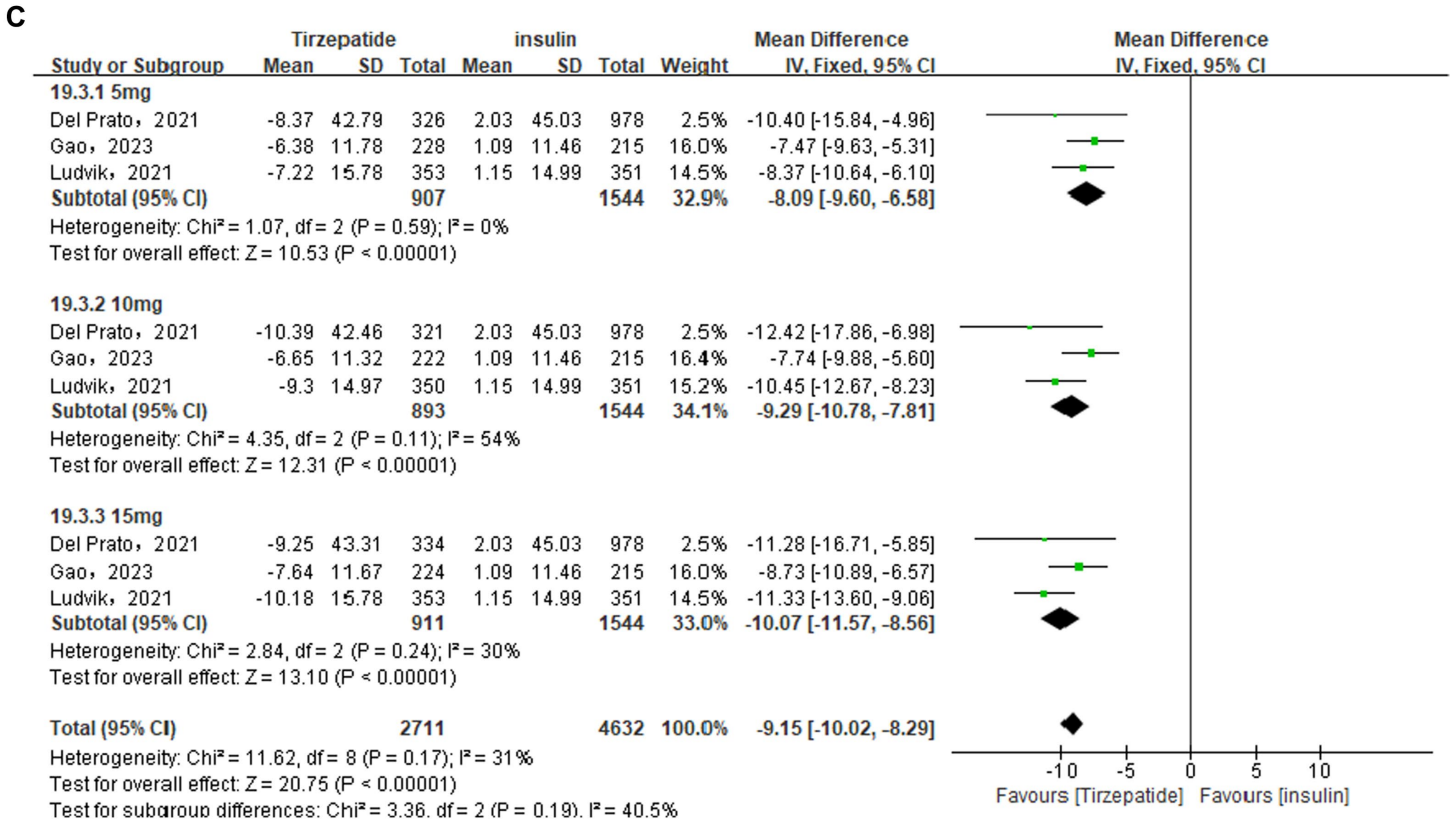
Forest diagram for tirzepatide vs GLP-1 RAs **(A)** and vs placebo **(B)** and vs insulin **(C)** for change in waist circumference (cm). GLP-1 RA: semaglutide and dulaglutide.

In this indicator analysis, 2 (26, 29), 6 (23, 24, 26, 27, 31, 32), and 3 (28, 30, 35) studies were separately included in different control groups, and were all analyzed using random effects models because of *I*_2_ > 50%. The results showed that the waist circumference of the tirzepatide group was significantly lower than that of the control group GLP-1 RA [MD = −4.08, 95% CI (−5.77, −2.39), *p* < 0.00001], placebo [MD = −7.71, 95% CI (−10.17, −5.25), *p* < 0.00001], insulin [MD = −9.15, 95% CI (−10.02, −8.29), *p* < 0.00001], all with statistical differences (Figure 5).

Sensitivity analysis showed that after removing the study Jastreboff et al. (23) in the placebo group, the subgroup statistical heterogeneity decreased from 93 to 75%, 97 to 0%, and 97 to 88%, respectively. The waist circumference of the tirzepatide group was still lower than that of the placebo group [MD = −6.03, 95% CI (−7.61, −4.45), *p* < 0.00001], and the difference was statistically significant. Likewise, sensitivity analysis were also performed in the GLP-1 group due to the high heterogeneity. The study Frias et al. (26) was removed and the statistical heterogeneity reduced from 67 to 51%. The results did not change.

### Body weight

**Figure 6:**
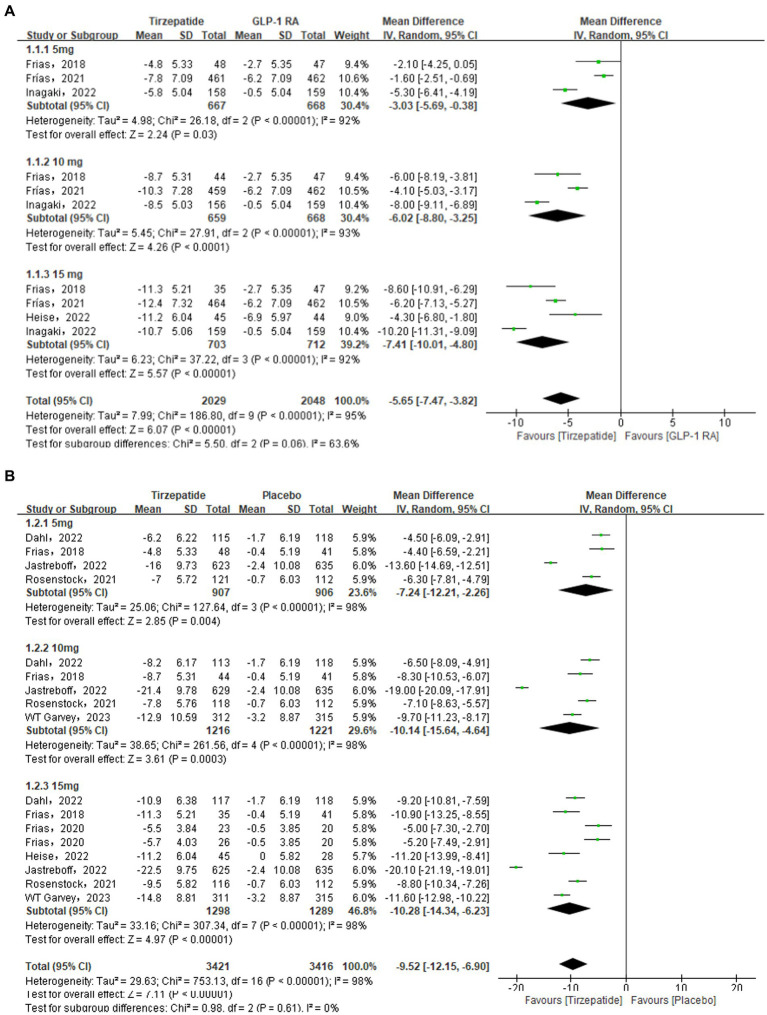

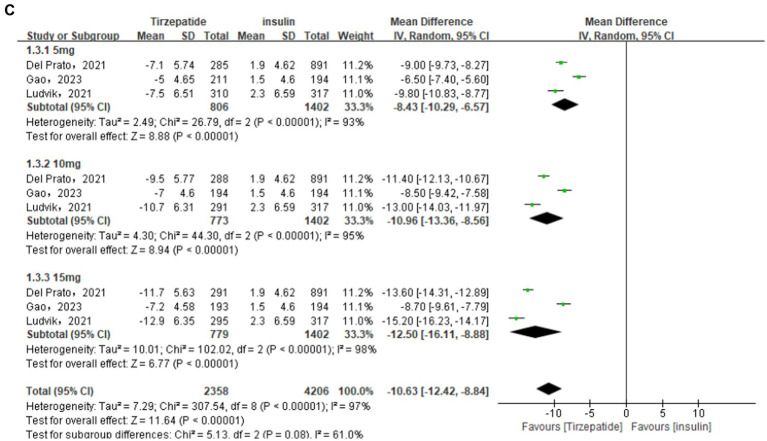
Forest diagram for tirzepatide vs GLP-1 RAs **(A)** and vs placebo **(B)** and vs insulin **(C)** for change in body weight (kg). GLP-1 RA: semaglutide and dulaglutide.

A total of 4 (26, 29, 33, 34) studies were included in comparing the tirzepatide group with the GLP-1 RAs group. According to the analysis’s findings, the tirzepatide group’s body weight was substantially lower than the GLP-1 RAs group’s [MD = −5.65, 95% CI (−7.47, −3.82), *p* < 0.001]. The results of subgroup analysis revealed that the tirzepatide group’s body weight was lower than that of the GLP-1 RAs group when they received doses of 5 mg [MD = −3.03, 95% CI (−5.69, −0.38), *p* = 0.03], 10 mg [MD = −6.02, 95% CI (−8.80, −3.25), *p* < 0.001], or 15 mg [MD = −7.41, 95% CI (−10.01, −4.80), *p* < 0.001]. There were a total of 7 (23, 24, 26, 27, 31–33) studies and 3 (28, 30, 35) studies were included in the placebo group and the insulin group. The analysis’s findings demonstrated that the tirzepatide group’s body weight were considerably lower than the placebo and the insulin group [MD = −9.52, 95% CI (−12.15, −6.90), *p* < 0.001; MD = −10.63, 95% CI (−12.42, −8.84), *p* < 0.001] (Figure 6).

### Weight loss ≥5, 10, 15, 20, 25%

Participants receiving different doses of tirzepatide showed a weight loss ≥5, 10%, or 15%, compared with GLP-1 RAs, placebo, and insulin in a meta-analysis. As shown in Table 2. The analysis results showed that the effective rate of the experimental group was significantly higher than that of the control group in weight loss. The differences were statistically significant. The tirzepatide group has a more outstanding advantage in weight loss ≥15% compared to weight loss ≥5 and 10%. Compared with placebo, the efficacy rate of tirzepatide in weight loss≥20%, 25% was much higher than that of placebo [RR = 30.43, 95% CI (19.56, 47.33), *p* < 0.001; RR = 37.25, 95% CI (26.03, 53.30), *p* < 0.001]. The effect size of weight loss≥20% or ≥25% was more than twice that of weight loss ≥15%.

**Table 2:** The results of meta-analysis for tirzepatide vs. control group for change in weight loss ≥5, 10, 15, 20, 25%.

Control	Intervention	Number of studies	Population	Effect model	RR (95%CI)	*p* value
**Weight loss ≥ 5%**						
GLP-1 RA	5 mg	3	703/1,348		2.24 [0.81, 6.15]	0.12
	10 mg	3	794/1,340		2.98 [0.95, 9.29]	0.06
	15 mg	3	827/1,350		2.99 [0.93, 9.61]	0.07
	Total effect	3	2,324/4,038	Random	2.61 [1.89, 3.60]	<0.00001
Placebo	5 mg	4	920/1,833		5.07 [2.85, 9.03]	<0.00001
	10 mg	5	1,350/2,462		4.31 [2.87, 6.47]	<0.00001
	15 mg	5	1,368/2,458		4.50 [2.99, 6.78]	<0.00001
	Total effect	5	3,638/6,753	Random	4.20 [3.44, 5.11]	<0.00001
Insulin	5 mg	3	677/2,451		9.03 [7.38, 11.05]	<0.00001
	10 mg	3	813/2,437		11.34 [9.30, 13.83]	<0.00001
	15 mg	3	873/2,455		12.11 [9.96, 14.73]	<0.00001
	Total effect	3	2,363/7,343	Fixed	10.83 [9.66, 12.15]	<0.00001
**Weight loss ≥ 10%**						
GLP-1 RA	5 mg	3	340/1,348		2.92 [0.78, 10.90]	0.11
	10 mg	3	434/1,340		4.89 [1.24, 19.24]	0.02
	15 mg	3	510/1,350		5.70 [1.36, 23.88]	0.02
	Total effect	3	1,284/4,038	Random	3.97 [2.54, 6.20]	<0.00001
Placebo	5 mg	4	618/1,833		12.31 [2.70, 56.15]	0.001
	10 mg	5	1,119/2,462		8.31 [2.24, 30.75]	0.002
	15 mg	5	998/2,458		9.17 [4.64, 18.12]	<0.00001
	Total effect	5	2,735/6,753	Random	9.91 [5.15, 19.09]	<0.00001
Insulin	5 mg	3	336/2,451		19.74 [13.17, 29.58]	<0.00001
	10 mg	3	484/2,437		29.47 [19.75, 43.98]	<0.00001
	15 mg	3	591/2,455		35.84 [24.13, 53.23]	<0.00001
	Total effect	3	1,153/7,343	Fixed	28.37 [22.53, 35.72]	<0.00001
**Weight loss ≥ 15%**						
GLP-1 RA	5 mg	3	137/1,348		4.50 [0.78, 25.83]	0.09
	10 mg	3	200/1,340		9.38 [1.48, 59.53]	0.02
	15 mg	3	290/1,350		13.83 [2.00, 95.78]	0.008
	Total effect	3	627/4,038	Random	6.22 [3.30, 11.72]	<0.00001
Placebo	5 mg	4	419/1,833		8.40 [6.28, 11.25]	<0.00001
	10 mg	5	732/2,462		12.97 [9.95, 16.91]	<0.00001
	15 mg	5	789/2,458		14.04 [10.77, 18.29]	<0.00001
	Total effect	5	1,940/6,753	Fixed	11.98 [10.24, 14.02]	<0.00001
Insulin	5 mg	3	117/2,451		38.31 [15.18, 96.64]	<0.00001
	10 mg	3	219/2,437		72.87 [28.76, 184.63]	<0.00001
	15 mg	3	317/2,455		104.47 [41.99, 259.92]	<0.00001
	Total effect	3	652/7,343	Fixed	72.01 [42.31, 122.53]	<0.00001
**Weight loss ≥ 20%**						
Placebo	5 mg	1	101/1,258		16.14 [7.12, 36.55]	<0.00001
	10 mg	2	238/1,891		33.32 [15.81, 70.21]	<0.00001
	15 mg	2	281/1,886		39.75 [18.89, 83.65]	<0.00001
	Total effect	2	620/5,035	Fixed	30.43 [19.56, 47.33]	<0.00001
**Weight loss ≥ 25%**						
Placebo	5 mg	1	205/1,258		25.10 [12.49, 50.45]	<0.00001
	10 mg	2	427/1,891		38.18 [21.13, 68.98]	<0.00001
	15 mg	2	500/1,886		45.13 [25.01, 81.45]	<0.00001
	Total effect	2	1,132/5,035	Fixed	37.25 [26.03, 53.30]	<0.00001

RR, Risk ratio; CI, confidence interval; GLP-1 RA, semaglutide and dulaglutide.

### Safety meta-analysis

Comparing the safety aspects of the tirzepatide group and the control group, meta-analysis showed that the total incidence of adverse events with tirzepatide was similar to the GLP-1 RA group [RR = 1.04, 95% CI (1.00, 1.09), *p* = 0.03], higher than the placebo group [RR = 1.11, 95% CI (1.05, 1.17), *p* = 0.0002] and the insulin group [RR = 1.20, 95% CI (1.12, 1.28), *p* < 0.00001], with a dose-dependent difference. In addition, There were statistically significant differences in the incidence of adverse events between tirzepatide and the control group. In this study, there was no significant difference in the incidence of serious adverse events between tirzepatide and placebo [RR = 0.93, 95% CI (0.77, 1.11), *p* = 0.42], but there was a significant statistical difference compared to GLP-1 RAs and insulin [RR = 0.81, 95% CI (0.70, 0.94), *p* = 0.005; RR = 0.29, 95% CI (0.12, 0.72), *p* = 0.007].

Compared with the placebo and insulin groups, the incidence of gastrointestinal adverse reactions in the tirzepatide group was significantly higher, and the differences were statistically significant. The main symptoms included nausea, diarrhea, vomiting, and decreased appetite. Compared with the GLP-1 RAs group, the risk of nausea, diarrhea, vomiting, and decreased appetite in the tirzepatide group was slightly higher. The incidence of pancreatitis in the tirzepatide group was similar to GLP-1 RAs, with slightly higher incidence of cholecystitis in the tirzepatide (10 mg, 15 mg) group compared to GLP-1 RAs. Compared with placebo, the incidence of pancreatitis and cholecystitis in the tirzepatide group was significantly higher. The incidence of pancreatitis in the tirzepatide (5 mg, 10 mg) group was higher than that in the insulin group, and the incidence of cholecystitis in the tirzepatide (10 mg) group was higher than that in the insulin group.

Compared to insulin, the hypoglycemic risk of tirzepatide was significantly lower, and the difference was statistically significant. Tirzepatide had a slightly higher risk of hypoglycemia than GLP-1 RAs and placebo, but the difference was not statistically significant. The risk of MACE-4 was higher for tirzepatide than for GLP-1 RAs, while the risk of MACE-4 was lower for tirzepatide compared to placebo and insulin. However, the differences were not statistically significant. The risk of hypersensitivity reactions between tirzepatide (5 mg, 15 mg) and GLP-1 RA, tirzepatide (10 mg, 15 mg) and placebo is equivalent. The risk of hypersensitivity reactions between tirzepatide and insulin is significantly higher, and it is dose-dependent. Compared with the control group, the tirzepatide group was lower in the risk of hypertension, while in the risk of lipase increased was higher. The incidence of neoplasms was lower in the tirzepatide group compared to the placebo group, but higher compared to the GLP-1 RA and insulin groups.

The above safety meta-analysis results are all shown in Table 3.

**Table 3:** The results of meta-analysis of safety.

Control	Intervention	Number of studies	RR (95% CI)	*p* value	*I*_2_ (%)	*p* value
**Total adverse events**						
GLP-1 RA	5 mg	3	1.01 [0.94, 1.08]	0.79	0	0.58
	10 mg	3	1.05 [0.98, 1.12]	0.19	0	0.59
	15 mg	4	1.07 [1.01, 1.14]	0.03	50	0.11
Placebo	5 mg	4	1.12 [1.06, 1.18]	<0.0001	0	0.46
	10 mg	5	1.08 [0.99, 1.18]	0.07	57	0.05
	15 mg	7	1.13 [1.01, 1.26]	0.03	73	0.001
Insulin	5 mg	3	1.13 [1.02, 1.25]	0.02	68	0.04
	10 mg	3	1.22 [1.06, 1.40]	0.004	85	0.002
	15 mg	3	1.26 [1.11, 1.41]	0.0002	81	0.006
**Serious adverse events**						
GLP-1 RA	5 mg	3	0.96 [0.27, 3.44]	0.95	79	0.009
	10 mg	3	0.14 [0.05, 0.33]	<0.0001	80	0.007
	15 mg	4	0.14 [0.05, 0.39]	0.0001	74	0.01
Placebo	5 mg	4	0.95 [0.66, 1.35]	0.76	0	0.83
	10 mg	5	0.98 [0.73, 1.32]	0.91	0	0.82
	15 mg	7	0.86 [0.63, 1.16]	0.33	0	0.65
Insulin	5 mg	3	0.84 [0.66, 1.06]	0.15	43	0.17
	10 mg	3	0.86 [0.67, 1.11]	0.25	0	0.85
	15 mg	3	0.73 [0.56, 0.96]	0.02	74	0.05
**Nausea**						
GLP-1 RA	5 mg	3	1.00 [0.69, 1.43]	0.22	35	0.22
	10 mg	3	1.25 [0.68, 2.31]	0.01	77	0.01
	15 mg	4	1.37 [0.94, 1.99]	0.08	56	0.08
Placebo	5 mg	4	2.66 [2.07, 3.42]	<0.00001	0	0.59
	10 mg	5	3.46 [2.79, 4.29]	<0.00001	0	0.61
	15 mg	7	3.35 [2.73, 4.11]	<0.00001	51	0.06
Insulin	5 mg	3	6.44 [4.30, 9.64]	<0.00001	0	0.54
	10 mg	3	10.44 [7.03, 15.49]	<0.00001	44	0.17
	15 mg	3	11.90 [8.13, 17.42]	<0.00001	0	0.59
**Diarrhea**						
GLP-1 RA	5 mg	3	1.37 [1.04, 1.82]	0.03	50	0.13
	10 mg	3	1.40 [1.06, 1.85]	0.02	0	0.97
	15 mg	4	1.25 [0.96, 1.62]	0.09	35	0.20
Placebo	5 mg	4	2.29 [1.76, 2.99]	<0.00001	53	0.10
	10 mg	5	2.47 [1.98, 3.08]	<0.00001	41	0.15
	15 mg	7	2.67 [2.17, 3.30]	<0.00001	50	0.06
Insulin	5 mg	3	5.67 [2.05, 15.69]	0.0008	87	0.0004
	10 mg	3	7.41 [2.85, 19.28]	<0.0001	86	0.0007
	15 mg	3	7.40 [2.96, 18.53]	<0.0001	85	0.001
**Vomiting**						
GLP-1 RA	5 mg	3	1.33 [0.38, 4.59]	0.65	76	0.02
	10 mg	3	1.45 [0.74, 2.83]	0.28	40	0.19
	15 mg	4	1.94 [0.76, 4.95]	0.17	74	0.009
Placebo	5 mg	4	4.04 [2.40, 6.82]	<0.00001	0	0.70
	10 mg	5	4.55 [3.02, 6.85]	<0.00001	0	0.41
	15 mg	7	5.54 [3.76, 8.14]	<0.00001	0	0.74
Insulin	5 mg	3	4.38 [2.59, 7.42]	<0.00001	0	0.43
	10 mg	3	7.22 [4.39, 11.87]	<0.00001	0	0.41
	15 mg	3	7.12 [4.35, 11.65]	<0.00001	0	0.53
**Decreased appetite**						
GLP-1 RA	5 mg	3	2.19 [1.14, 4.19]	0.02	50	0.14
	10 mg	3	2.33 [1.11, 4.86]	0.02	60	0.08
	15 mg	4	2.06 [0.84, 5.03]	0.11	89	<0.00001
Placebo	5 mg	4	3.35 [2.16, 5.20]	<0.00001	0	0.63
	10 mg	5	4.37 [3.00, 6.37]	<0.00001	0	0.59
	15 mg	7	3.71 [2.65, 5.18]	<0.00001	20	0.28
Insulin	5 mg	3	27.82 [12.15, 63.69]	<0.00001	48	0.15
	10 mg	3	39.92 [17.39, 91.68]	<0.00001	51	0.13
	15 mg	3	38.91 [16.97, 89.21]	<0.00001	47	0.15
**Pancreatitis**						
GLP-1 RA	5 mg	3	0.74 [0.17, 3.31]	0.70	63	0.10
	10 mg	3	0.67 [0.11, 3.97]	0.66	–	–
	15 mg	4	1.00 [0.23, 4.37]	1.00	0	0.42
Placebo	5 mg	2	8.01 [1.46, 44.01]	0.07	61	0.11
	10 mg	3	5.02 [1.46, 17.23]	0.06	63	0.07
	15 mg	4	3.16 [1.09, 9.21]	0.08	43	0.15
Insulin	5 mg	2	2.47 [0.63, 9.71]	0.20	65	0.09
	10 mg	2	1.73 [0.38, 7.87]	0.48	53	0.14
	15 mg	2	0.98 [0.16, 6.05]	0.98	8	0.30
**Cholecystitis**						
GLP-1 RA	5 mg	2	0.99 [0.14, 6.95]	0.99	0	0.34
	10 mg	2	2.40 [0.35, 16.27]	0.37	0	0.45
	15 mg	2	1.51 [0.25, 9.04]	0.65	31	0.23
Placebo	5 mg	2	2.04 [0.51, 8.13]	0.31	–	–
	10 mg	3	1.93 [0.69, 5.39]	0.21	0	0.47
	15 mg	4	1.42 [0.45, 4.47]	0.54	0	0.93
Insulin	5 mg	3	0.23 [0.01, 4.13]	0.32	–	–
	10 mg	3	1.29 [0.32, 5.22]	0.72	0	0.56
	15 mg	3	0.87 [0.19, 3.85]	0.85	0	0.38
**Hypoglycemia (<70 mg/dL)**						
GLP-1 RA	5 mg	2	1.35 [0.31, 5.86]	0.69	0	0.33
	10 mg	2	2.77 [0.75, 10.16]	0.13	0	0.93
	15 mg	2	3.50 [0.92, 13.34]	0.07	6	0.30
Placebo	5 mg	3	1.09 [0.89, 1.35]	0.40	52	0.13
	10 mg	3	1.16 [0.95, 1.43]	0.14	63	0.07
	15 mg	4	1.18 [0.95, 1.46]	0.13	65	0.03
Insulin	5 mg	3	0.39 [0.21, 0.73]	0.003	95	<0.00001
	10 mg	3	0.48 [0.30, 0.77]	0.002	92	<0.00001
	15 mg	3	0.50 [0.33, 0.76]	0.001	91	<0.00001
**MACE-4**						
GLP-1 RA	5 mg	3	2.20 [0.49, 9.77]	0.30	58	0.12
	10 mg	3	1.51 [0.26, 8.91]	0.65	–	–
	15 mg	4	1.33 [0.30, 5.91]	0.71	63	0.10
Placebo	5 mg	4	0.56 [0.19, 1.66]	0.30	0	0.64
	10 mg	5	0.84 [0.37, 1.90]	0.68	0	0.78
	15 mg	7	0.36 [0.14, 0.96]	0.04	0	0.63
Insulin	5 mg	3	0.99 [0.63, 1.58]	0.98	0	0.63
	10 mg	3	0.85 [0.52, 1.39]	0.53	0	0.97
	15 mg	3	0.60 [0.34, 1.06]	0.08	38	0.20
**Hypersensitivity**						
GLP-1 RA	5 mg	3	1.00 [0.44, 2.24]	0.99	22	0.26
	10 mg	3	1.36 [0.64, 2.88]	0.43	0	0.34
	15 mg	3	1.00 [0.45, 2.21]	1.00	3	0.36
Placebo	5 mg	4	1.42 [0.62, 3.24]	0.41	51	0.13
	10 mg	4	0.89 [0.36, 2.22]	0.80	0	0.58
	15 mg	5	0.92 [0.41, 2.06]	0.85	3	0.39
Insulin	5 mg	3	1.75 [0.83, 3.68]	0.14	0	0.45
	10 mg	3	1.77 [0.83, 3.80]	0.14	0	0.36
	15 mg	3	1.96 [0.93, 4.15]	0.08	0	0.84
**Hypertension**						
GLP-1 RA	5 mg	3	0.30 [0.08, 1.07]	0.06	0	0.83
	10 mg	3	0.20 [0.04, 0.93]	0.04	0	0.94
	15 mg	3	0.20 [0.04, 0.91]	0.04	0	0.74
Placebo	5 mg	3	0.37 [0.12, 1.14]	0.08	0	0.89
	10 mg	3	0.37 [0.12, 1.13]	0.08	0	0.90
	15 mg	3	0.30 [0.08, 1.08]	0.07	0	0.50
Insulin	5 mg	2	0.54 [0.27, 1.09]	0.09	0	0.70
	10 mg	2	0.40 [0.18, 0.86]	0.02	56	0.13
	15 mg	2	0.54 [0.27, 1.09]	0.08	0	0.71
**Lipase increased**						
GLP-1 RA	5 mg	3	1.66 [0.74, 3.70]	0.22	42	0.19
	10 mg	3	1.13 [0.48, 2.68]	0.78	0	0.52
	15 mg	4	1.45 [0.68, 3.07]	0.34	39	0.20
Placebo	5 mg	2	1.37 [0.45, 4.19]	0.58	0	0.49
	10 mg	2	1.20 [0.38, 3.83]	0.75	0	0.82
	15 mg	3	1.97 [0.83, 4.67]	0.12	51	0.13
Insulin	5 mg	3	3.06 [1.92, 4.87]	<0.00001	46	0.16
	10 mg	3	3.05 [1.92, 4.82]	<0.00001	26	0.26
	15 mg	3	3.64 [2.35, 5.63]	<0.00001	0	0.69
**Neoplasms benign, malignant, and unspecified**						
GLP-1 RA	5 mg	3	4.00 [0.85, 18.73]	0.08	0	0.75
	10 mg	3	2.01 [0.37, 10.90]	0.42	0	1.00
	15 mg	3	2.49 [0.49, 12.76]	0.27	0	0.81
Placebo	5 mg	4	0.68 [0.29, 1.63]	0.39	0	0.74
	10 mg	5	0.25 [0.09, 0.67]	0.06	0	0.86
	15 mg	5	0.52 [0.25, 1.08]	0.08	0	0.96
Insulin	5 mg	3	1.10 [0.47, 2.54]	0.83	15	0.31
	10 mg	3	1.47 [0.68, 3.14]	0.33	0	0.41
	15 mg	3	1.25 [0.56, 2.81]	0.58	43	0.17

RR, Risk ratio; CI, confidence interval; GLP-1 RA, semaglutide and dulaglutide; MACE-4, Major Adverse Cardiovascular Events-4.

## Discussion

This study conducted a meta-analysis of the included literature to systematically evaluate the efficacy and safety of tirzepatide as a weight loss drug. The forest diagram showed that tirzepatide significantly reduced BMI, waist circumference and body weight compared to the control group GLP-1 RAs, placebo, and insulin. And tirzepatide has a significant advantage in weight loss ≥20%, 25% compared with placebo. It has been shown that tirzepatide can reduce weight in a dose-dependent manner. Compared with GLP-1 RAs, placebo and insulin, tirzepatide can significantly reduce the HbA1c level of patients, and the results have been demonstrated in other studies (36, 37). This indicates that tirzepatide has a better metabolic effect. The dual activation of tirzepatide with GIP and GLP-1 receptors leads to an increase in insulin secretion and peripheral insulin sensitivity, while slowing down gastric emptying and the neuromodulation of gastrointestinal motility (19). According to reports, compared to the GLP-1 receptor, tirzepatide has a stronger interaction with the GIP receptor, making the drug an unbalanced and biased dual GIP and GLP-1 receptor agonist (15). In some mouse studies, compared to unbiased agonists, GLP-1 receptor agonists with similar bias signals have better effects on blood glucose and weight control (38, 39).

In terms of safety, the total incidence of adverse reactions with tirzepatide was similar to that of the GLP-1 RA group, slightly higher than that of the placebo and insulin groups. However, there was no significant difference in the incidence of serious adverse events compared to the placebo group. Gastrointestinal adverse reactions were the most common adverse reactions of tirzepatide, with a higher incidence compared to the placebo and insulin groups and slightly higher to the GLP-1 RA group. Tirzepatide gastrointestinal adverse reactions mainly included nausea, vomiting, diarrhea, and decreased appetite, but the severity of these symptoms was mostly mild or moderate and the duration is relatively short. These effects are usually observed in irritable bowel syndrome and may suggest a potential effect of tirzepatide on the gut microbiota (40). The risk of hypoglycemia is significantly lower in the insulin group, slightly higher to the GLP-1 RA and placebo groups but without statistically significant differences.

However, in this study, the difference in the incidence of other adverse events such as pancreatitis, cholecystitis, MACE-4, hypersensitivity reactions and neoplasms between the two groups is not statistically significant, but the study of these adverse events of tirzepatide has important clinical significance. It serves as a warning for high-risk populations and adopts avoiding or monitoring the use of tirzepatide in order to reduce the incidence of drug-induced diseases or the mortality caused by them. The occurrence of these adverse events may be related to the widespread distribution of GLP-1 receptors in multiple organs or tissues such as the pancreas, heart, and blood vessels, which are simultaneously affected when blood sugar rapidly decreases (41). According to Sattar et al. (42) in a meta-analysis of cardiovascular event risk assessment for tirzepatide, compared with other hypoglycemic drugs, insulin and placebo, tirzepatide does not increase the risk of major cardiovascular events in patients with type 2 diabetes. The ongoing RCT (43–46) will confirm whether tirzepatide has long-term safety, and SURPASS-CVOT (NCT04255433) will provide more data on cardiovascular safety for tirzepatide.

Recent studies have shown that obesity is now recognized as comprising many different phenotypes rather than being considered as a singular disease (47). Metabolically healthy obese/overweight (MHO) is a unique phenotype, which is best defined as being obese or overweight but not have any major metabolic disorder or cardiovascular diseases like T2DM. However, it should not be considered a benign disease. MHO patients are at high risk of transforming into metabolically unhealthy obesity, such as diabetic obesity. MHO is also being recognized as a significant risk factor for the development of cardiovascular, cerebrovascular, and peripheral artery disease (48). Therefore, it is also critical to focus on weight loss treatment in the MHO population. This study involved two types of study patients, namely MHO patients and diabetic obese patients. Tirzepatide has a different mechanism of action for these two groups of patients. The effects of Tirzepatide on MHO patients were mainly reflected in improving insulin sensitivity, promoting glucose uptake and utilization, inhibiting fatty acid synthesis in adipocytes and reducing fat synthesis (49). For metabolically unhealthy obese patients, Tirzepatide, in addition to improving insulin resistance and inhibiting fat synthesis, also treats this type of obesity by regulating lipid metabolism, lowering blood pressure and improving cardiovascular health (49). Given the beneficial results of Tirzepatide in metabolic parameters and weight loss in obese patients, Tirzepatide is a potential candidate for use not only in metabolically unhealthy obese patients, but also in MHO patients to help reduce the risk of adverse cardiovascular outcomes and conversion to metabolically unhealthy phenotypes.

Comparing this study with the meta-analysis of tirzepatide conducted by Lin et al. (37), this study adds two new high-quality RCT study (24, 35) with almost participants from the Asia Pacific region, which increase the reliability and applicability of the research results. The latter only analyzes the body weight, one of the efficacy indicators of weight-loss drug, while this study also includes BMI, waist circumference, which are also important indicators of cardiovascular disease and type 2 diabetes. In terms of weight loss percentage, this study focuses on emphasizing the significant weight loss trend of tirzepatide in weight loss ≥20, 25%. In terms of safety, this study also analyzed severe prognostic factors such as pancreatitis, cholecystitis, MACE-4, hypersensitivity reactions, hypertension, lipase increased and neoplasms. Comparing this study with the study of Tan et al. (50), the latter included seven studies, but one of them had no control group and was not included in the analysis. Therefore, this study included six more RCT studies (24, 27, 31, 32, 34, 35) than the latter. The number of patients in this study reached tens of thousands for the first time, and the results were more reliable. The analysis of weight loss ≥20, 25% was added in this study. In terms of efficacy indicators, the analysis of BMI was added in this study. Pancreatitis, cholecystitis, MACE-4, hypersensitivity reactions, hypertension, and lipase increased were added in terms of safety indicators.

There are several limitations. First, China has a large population and accounting for more than a quarter of all diabetes patients in the world (51). However, only three of the 12 articles included mainly studied on people in the Asia Pacific region, and further data on the efficacy and safety of tirzepatide in the broader Asian patient population are needed. Second, most of the subjects included in this study were patients with type 2 diabetes, and the study on the use of tirzepatide in MHO patients was more consistent with its original intention as a weight loss drug. Third, the research subjects are all adults, and it is still necessary to study the safety of tirzepatide for use in children and adolescents. Finally, the current research duration is relatively limited, more high-quality, large-scale clinical studies are still needed to verify and promote clinical use. And the long-term safety of tirzepatide still needs to be investigated.

At present, FDA has only approved five drugs as alternatives to obesity treatment (orlistat, phentermine/topiramate, naltrexone/bupropion, liraglutide 3 mg and semaglutide 2.4 mg) (52). As more and more literature shows that tirzepatide has a good effect on weight and has good safety, tirzepatide has the potential to become a new weight loss drug, and more high-quality RCTs will be used to verify this indication in the future.

## Data availability statement

The original contributions presented in the study are included in the article/supplementary material, further inquiries can be directed to the corresponding author.

## Author contributions

WC: Data curation, Writing – original draft. RZ: Data curation, Writing – original draft. YY: Data curation, Writing – original draft. QW: Data curation, Writing – review & editing. JZ: Data curation, Writing – review & editing.
